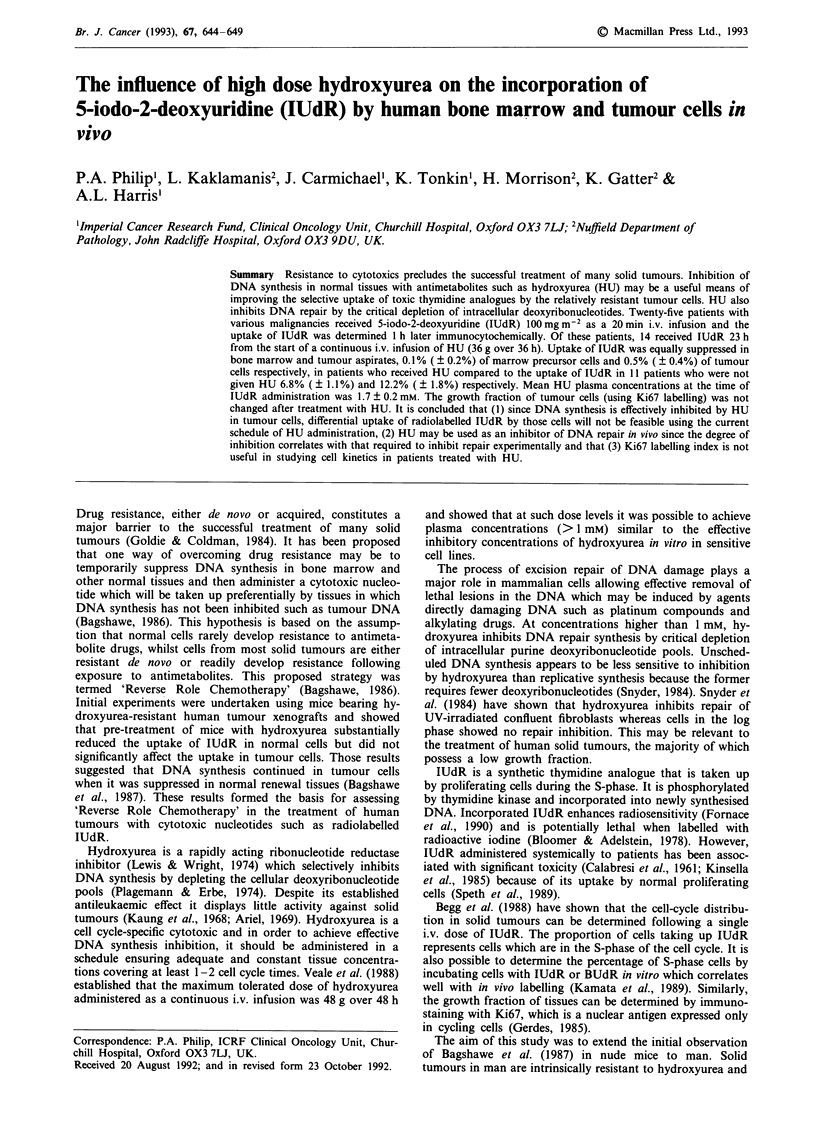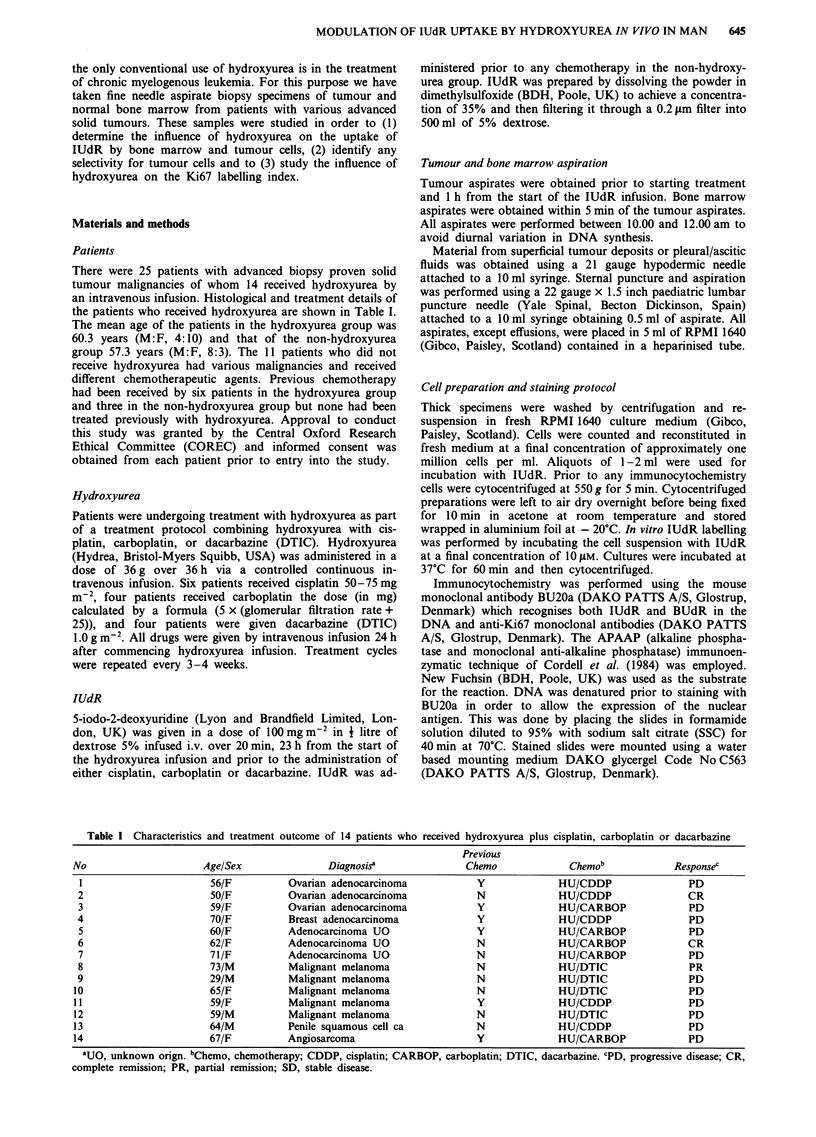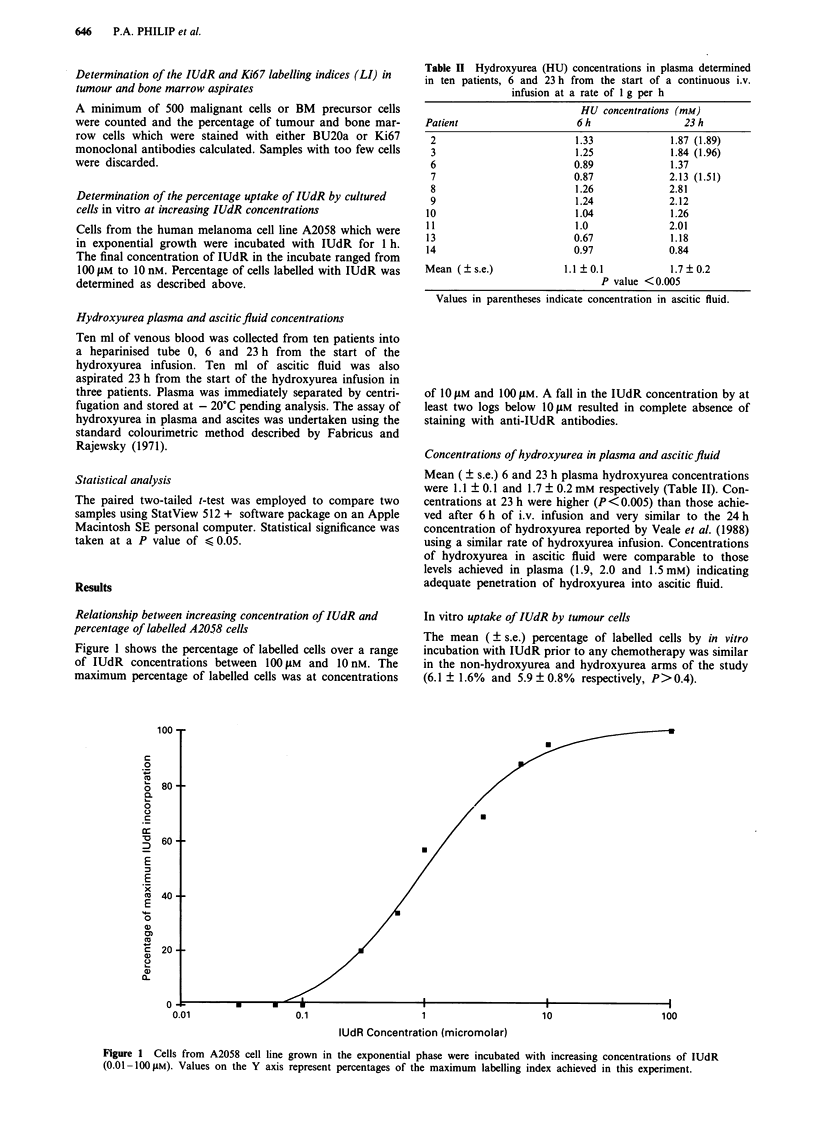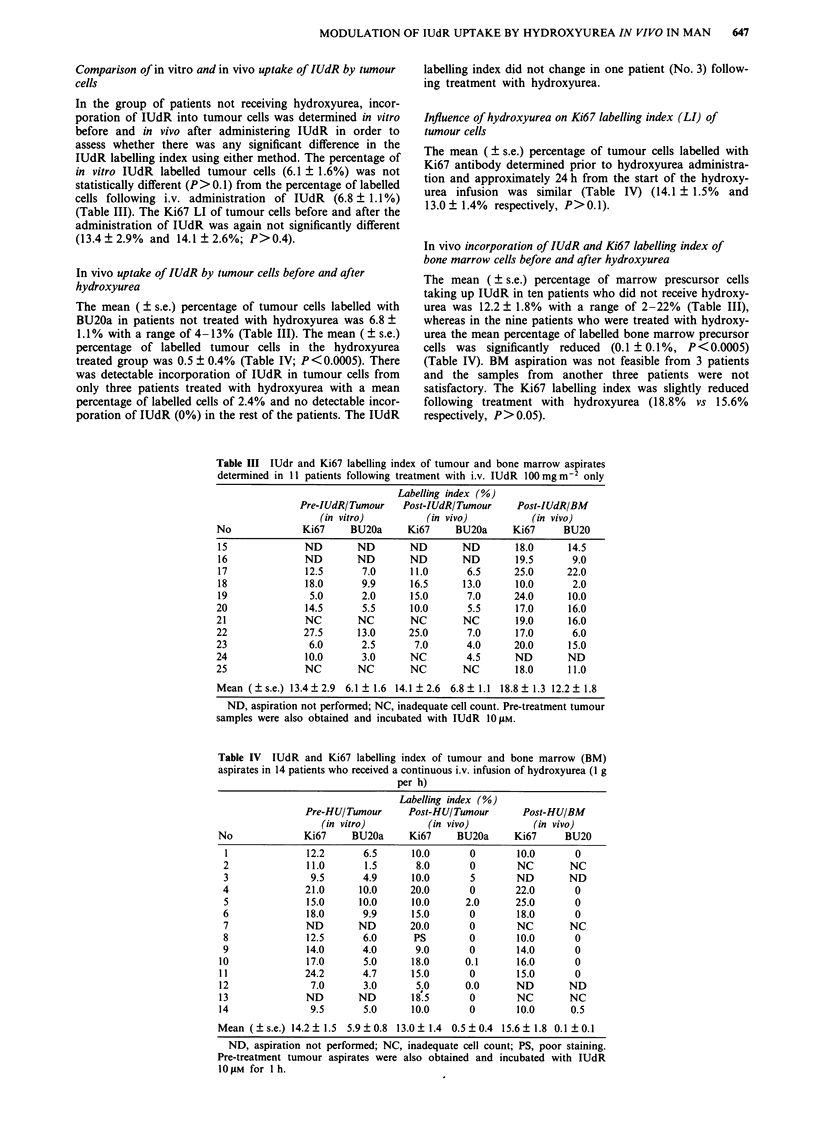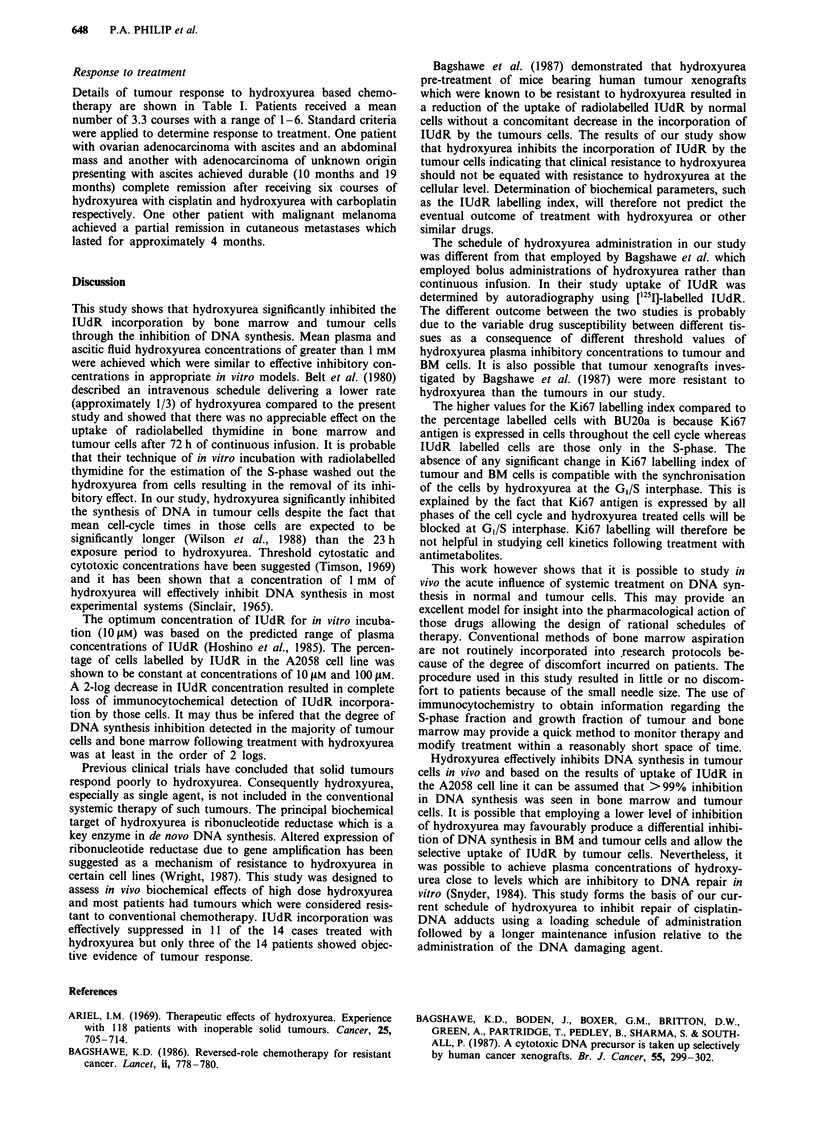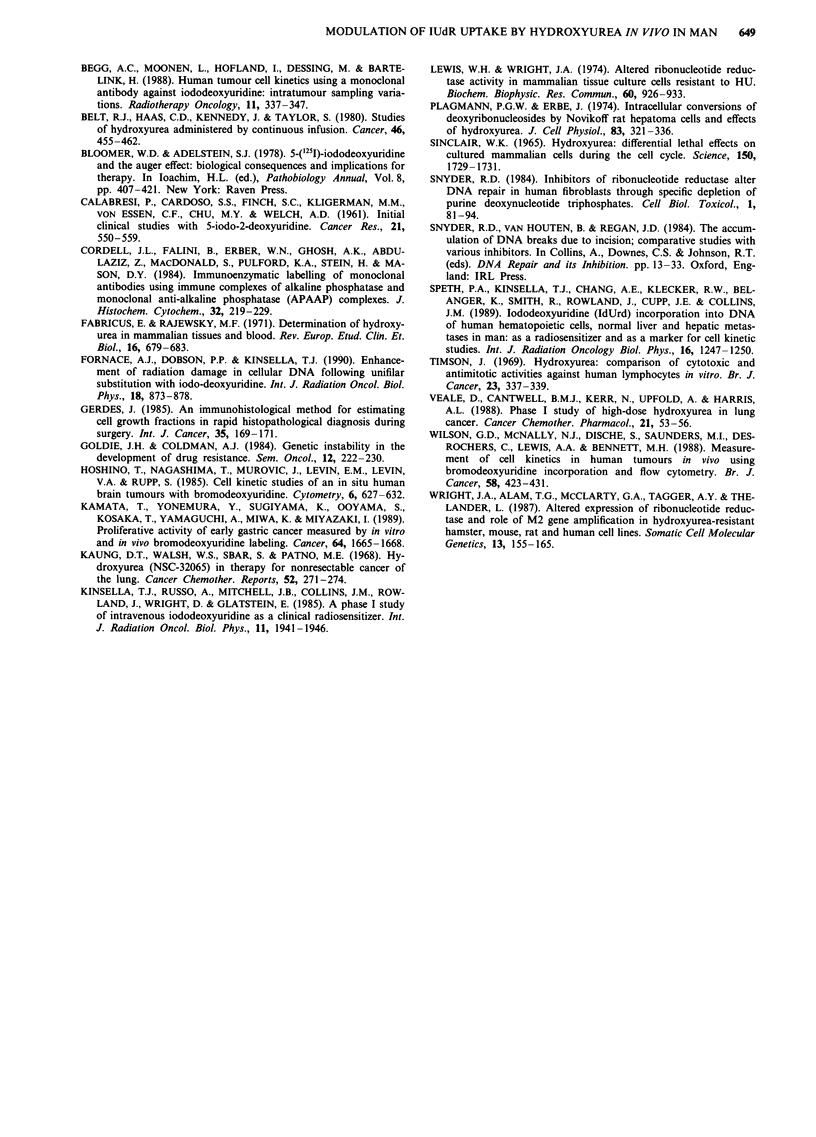# The influence of high dose hydroxyurea on the incorporation of 5-iodo-2-deoxyuridine (IUdR) by human bone marrow and tumour cells in vivo.

**DOI:** 10.1038/bjc.1993.120

**Published:** 1993-04

**Authors:** P. A. Philip, L. Kaklamanis, J. Carmichael, K. Tonkin, H. Morrison, K. Gatter, A. L. Harris

**Affiliations:** Imperial Cancer Research Fund, Clinical Oncology Unit, Churchill Hospital, Oxford, UK.

## Abstract

Resistance to cytotoxics precludes the successful treatment of many solid tumours. Inhibition of DNA synthesis in normal tissues with antimetabolites such as hydroxyurea (HU) may be a useful means of improving the selective uptake of toxic thymidine analogues by the relatively resistant tumour cells. HU also inhibits DNA repair by the critical depletion of intracellular deoxyribonucleotides. Twenty-five patients with various malignancies received 5-iodo-2-deoxyuridine (IUdR) 100 mg m-2 as a 20 min i.v. infusion and the uptake of IUdR was determined 1 h later immunocytochemically. Of these patients, 14 received IUdR 23 h from the start of a continuous i.v. infusion of HU (36 g over 36 h). Uptake of IUdR was equally suppressed in bone marrow and tumour aspirates, 0.1% (+/- 0.2%) of marrow precursor cells and 0.5% (+/- 0.4%) of tumour cells respectively, in patients who received HU compared to the uptake of IUdR in 11 patients who were not given HU 6.8% (+/- 1.1%) and 12.2% (+/- 1.8%) respectively. Mean HU plasma concentrations at the time of IUdR administration was 1.7 +/- 0.2 mM. The growth fraction of tumour cells (using Ki67 labelling) was not changed after treatment with HU. It is concluded that (1) since DNA synthesis is effectively inhibited by HU in tumour cells, differential uptake of radiolabelled IUdR by those cells will not be feasible using the current schedule of HU administration, (2) HU may be used as an inhibitor of DNA repair in vivo since the degree of inhibition correlates with that required to inhibit repair experimentally and that (3) Ki67 labelling index is not useful in studying cell kinetics in patients treated with HU.


Br. J. Cancer (1993), 67, 644–649 © Macmillan Press Ltd., 1993

### The influence of high dose hydroxyurea on the incorporation of 5-iodo-2-deoxyuridine (IUdR) by human bone marrow and tumour cells *in vivo*

P.A. Philip[1], L. Kaklamanis[2], J. Carmichael[1], K. Tonkin[1], H. Morrison[2], K. Gatter[2] & A.L. Harris[1]

[1]*Imperial Cancer Research Fund, Clinical Oncology Unit, Churchill Hospital, Oxford OX3 7LJ;* [2]*Nuffield Department of Pathology, John Radcliffe Hospital, Oxford OX3 9DU, UK.*

**Summary** Resistance to cytotoxics precludes the successful treatment of many solid tumours. Inhibition of DNA synthesis in normal tissues with antimetabolites such as hydroxyurea (HU) may be a useful means of improving the selective uptake of toxic thymidine analogues by the relatively resistant tumour cells. HU also inhibits DNA repair by the critical depletion of intracellular deoxyribonucleotides. Twenty-five patients with various malignancies received 5-iodo-2-deoxyuridine (IUdR) 100 mg m$^{-2}$ as a 20 min i.v. infusion and the uptake of IUdR was determined 1 h later immunocytochemically. Of these patients, 14 received IUdR 23 h from the start of a continuous i.v. infusion of HU (36 g over 36 h). Uptake of IUdR was equally suppressed in bone marrow and tumour aspirates, 0.1% (± 0.2%) of marrow precursor cells and 0.5% (± 0.4%) of tumour cells respectively, in patients who received HU compared to the uptake of IUdR in 11 patients who were not given HU 6.8% (± 1.1%) and 12.2% (± 1.8%) respectively. Mean HU plasma concentrations at the time of IUdR administration was 1.7 ± 0.2 mM. The growth fraction of tumour cells (using Ki67 labelling) was not changed after treatment with HU. It is concluded that (1) since DNA synthesis is effectively inhibited by HU in tumour cells, differential uptake of radiolabelled IUdR by those cells will not be feasible using the current schedule of HU administration, (2) HU may be used as an inhibitor of DNA repair *in vivo* since the degree of inhibition correlates with that required to inhibit repair experimentally and that (3) Ki67 labelling index is not useful in studying cell kinetics in patients treated with HU.

Drug resistance, either *de novo* or acquired, constitutes a major barrier to the successful treatment of many solid tumours (Goldie & Coldman, 1984). It has been proposed that one way of overcoming drug resistance may be to temporarily suppress DNA synthesis in bone marrow and other normal tissues and then administer a cytotoxic nucleotide which will be taken up preferentially by tissues in which DNA synthesis has not been inhibited such as tumour DNA (Bagshawe, 1986). This hypothesis is based on the assumption that normal cells rarely develop resistance to antimetabolite drugs, whilst cells from most solid tumours are either resistant *de novo* or readily develop resistance following exposure to antimetabolites. This proposed strategy was termed 'Reverse Role Chemotherapy' (Bagshawe, 1986). Initial experiments were undertaken using mice bearing hydroxyurea-resistant human tumour xenografts and showed that pre-treatment of mice with hydroxyurea substantially reduced the uptake of IUdR in normal cells but did not significantly affect the uptake in tumour cells. Those results suggested that DNA synthesis continued in tumour cells when it was suppressed in normal renewal tissues (Bagshawe *et al.*, 1987). These results formed the basis for assessing 'Reverse Role Chemotherapy' in the treatment of human tumours with cytotoxic nucleotides such as radiolabelled IUdR.

Hydroxyurea is a rapidly acting ribonucleotide reductase inhibitor (Lewis & Wright, 1974) which selectively inhibits DNA synthesis by depleting the cellular deoxyribonucleotide pools (Plagemann & Erbe, 1974). Despite its established antileukaemic effect it displays little activity against solid tumours (Kaung *et al.*, 1968; Ariel, 1969). Hydroxyurea is a cell cycle-specific cytotoxic and in order to achieve effective DNA synthesis inhibition, it should be administered in a schedule ensuring adequate and constant tissue concentrations covering at least 1–2 cell cycle times. Veale *et al.* (1988) established that the maximum tolerated dose of hydroxyurea administered as a continuous i.v. infusion was 48 g over 48 h and showed that at such dose levels it was possible to achieve plasma concentrations (> 1 mM) similar to the effective inhibitory concentrations of hydroxyurea *in vitro* in sensitive cell lines.

The process of excision repair of DNA damage plays a major role in mammalian cells allowing effective removal of lethal lesions in the DNA which may be induced by agents directly damaging DNA such as platinum compounds and alkylating drugs. At concentrations higher than 1 mM, hydroxyurea inhibits DNA repair synthesis by critical depletion of intracellular purine deoxyribonucleotide pools. Unscheduled DNA synthesis appears to be less sensitive to inhibition by hydroxyurea than replicative synthesis because the former requires fewer deoxyribonucleotides (Snyder, 1984). Snyder *et al.* (1984) have shown that hydroxyurea inhibits repair of UV-irradiated confluent fibroblasts whereas cells in the log phase showed no repair inhibition. This may be relevant to the treatment of human solid tumours, the majority of which possess a low growth fraction.

IUdR is a synthetic thymidine analogue that is taken up by proliferating cells during the S-phase. It is phosphorylated by thymidine kinase and incorporated into newly synthesised DNA. Incorporated IUdR enhances radiosensitivity (Fornace *et al.*, 1990) and is potentially lethal when labelled with radioactive iodine (Bloomer & Adelstein, 1978). However, IUdR administered systemically to patients has been associated with significant toxicity (Calabresi *et al.*, 1961; Kinsella *et al.*, 1985) because of its uptake by normal proliferating cells (Speth *et al.*, 1989).

Begg *et al.* (1988) have shown that the cell-cycle distribution in solid tumours can be determined following a single i.v. dose of IUdR. The proportion of cells taking up IUdR represents cells which are in the S-phase of the cell cycle. It is also possible to determine the percentage of S-phase cells by incubating cells with IUdR or BUdR *in vitro* which correlates well with *in vivo* labelling (Kamata *et al.*, 1989). Similarly, the growth fraction of tissues can be determined by immunostaining with Ki67, which is a nuclear antigen expressed only in cycling cells (Gerdes, 1985).

The aim of this study was to extend the initial observation of Bagshawe *et al.* (1987) in nude mice to man. Solid tumours in man are intrinsically resistant to hydroxyurea and

Correspondence: P.A. Philip, ICRF Clinical Oncology Unit, Churchill Hospital, Oxford OX3 7LJ, UK.
Received 20 August 1992; and in revised form 23 October 1992.

the only conventional use of hydroxyurea is in the treatment of chronic myelogenous leukemia. For this purpose we have taken fine needle aspirate biopsy specimens of tumour and normal bone marrow from patients with various advanced solid tumours. These samples were studied in order to (1) determine the influence of hydroxyurea on the uptake of IUdR by bone marrow and tumour cells, (2) identify any selectivity for tumour cells and to (3) study the influence of hydroxyurea on the Ki67 labelling index.

### Materials and methods

### *Patients*

There were 25 patients with advanced biopsy proven solid tumour malignancies of whom 14 received hydroxyurea by an intravenous infusion. Histological and treatment details of the patients who received hydroxyurea are shown in Table I. The mean age of the patients in the hydroxyurea group was 60.3 years (M:F, 4:10) and that of the non-hydroxyurea group 57.3 years (M:F, 8:3). The 11 patients who did not receive hydroxyurea had various malignancies and received different chemotherapeutic agents. Previous chemotherapy had been received by six patients in the hydroxyurea group and three in the non-hydroxyurea group but none had been treated previously with hydroxyurea. Approval to conduct this study was granted by the Central Oxford Research Ethical Committee (COREC) and informed consent was obtained from each patient prior to entry into the study.

### *Hydroxyurea*

Patients were undergoing treatment with hydroxyurea as part of a treatment protocol combining hydroxyurea with cisplatin, carboplatin, or dacarbazine (DTIC). Hydroxyurea (Hydrea, Bristol-Myers Squibb, USA) was administered in a dose of 36 g over 36 h via a controlled continuous intravenous infusion. Six patients received cisplatin 50–75 mg $m^{-2}$, four patients received carboplatin the dose (in mg) calculated by a formula (5 × (glomerular filtration rate + 25)), and four patients were given dacarbazine (DTIC) 1.0 g $m^{-2}$. All drugs were given by intravenous infusion 24 h after commencing hydroxyurea infusion. Treatment cycles were repeated every 3–4 weeks.

### *IUdR*

5-iodo-2-deoxyuridine (Lyon and Brandfield Limited, London, UK) was given in a dose of 100 mg $m^{-2}$ in ½ litre of dextrose 5% infused i.v. over 20 min, 23 h from the start of the hydroxyurea infusion and prior to the administration of either cisplatin, carboplatin or dacarbazine. IUdR was administered prior to any chemotherapy in the non-hydroxyurea group. IUdR was prepared by dissolving the powder in dimethylsulfoxide (BDH, Poole, UK) to achieve a concentration of 35% and then filtering it through a 0.2 μm filter into 500 ml of 5% dextrose.

### *Tumour and bone marrow aspiration*

Tumour aspirates were obtained prior to starting treatment and 1 h from the start of the IUdR infusion. Bone marrow aspirates were obtained within 5 min of the tumour aspirates. All aspirates were performed between 10.00 and 12.00 am to avoid diurnal variation in DNA synthesis.

Material from superficial tumour deposits or pleural/ascitic fluids was obtained using a 21 gauge hypodermic needle attached to a 10 ml syringe. Sternal puncture and aspiration was performed using a 22 gauge × 1.5 inch paediatric lumbar puncture needle (Yale Spinal, Becton Dickinson, Spain) attached to a 10 ml syringe obtaining 0.5 ml of aspirate. All aspirates, except effusions, were placed in 5 ml of RPMI 1640 (Gibco, Paisley, Scotland) contained in a heparinised tube.

### *Cell preparation and staining protocol*

Thick specimens were washed by centrifugation and resuspension in fresh RPMI 1640 culture medium (Gibco, Paisley, Scotland). Cells were counted and reconstituted in fresh medium at a final concentration of approximately one million cells per ml. Aliquots of 1–2 ml were used for incubation with IUdR. Prior to any immunocytochemistry cells were cytocentrifuged at 550 *g* for 5 min. Cytocentrifuged preparations were left to air dry overnight before being fixed for 10 min in acetone at room temperature and stored wrapped in aluminium foil at − 20°C. *In vitro* IUdR labelling was performed by incubating the cell suspension with IUdR at a final concentration of 10 μM. Cultures were incubated at 37°C for 60 min and then cytocentrifuged.

Immunocytochemistry was performed using the mouse monoclonal antibody BU20a (DAKO PATTS A/S, Glostrup, Denmark) which recognises both IUdR and BUdR in the DNA and anti-Ki67 monoclonal antibodies (DAKO PATTS A/S, Glostrup, Denmark). The APAAP (alkaline phosphatase and monoclonal anti-alkaline phosphatase) immunoenzymatic technique of Cordell *et al.* (1984) was employed. New Fuchsin (BDH, Poole, UK) was used as the substrate for the reaction. DNA was denatured prior to staining with BU20a in order to allow the expression of the nuclear antigen. This was done by placing the slides in formamide solution diluted to 95% with sodium salt citrate (SSC) for 40 min at 70°C. Stained slides were mounted using a water based mounting medium DAKO glycergel Code No C563 (DAKO PATTS A/S, Glostrup, Denmark).

**Table I** Characteristics and treatment outcome of 14 patients who received hydroxyurea plus cisplatin, carboplatin or dacarbazine

| *No* | *Age/Sex* | *Diagnosis*[a] | *Previous Chemo* | *Chemo*[b] | *Response*[c] |
|---|---|---|---|---|---|
| 1 | 56/F | Ovarian adenocarcinoma | Y | HU/CDDP | PD |
| 2 | 50/F | Ovarian adenocarcinoma | N | HU/CDDP | CR |
| 3 | 59/F | Ovarian adenocarcinoma | Y | HU/CARBOP | PD |
| 4 | 70/F | Breast adenocarcinoma | Y | HU/CDDP | PD |
| 5 | 60/F | Adenocarcinoma UO | Y | HU/CARBOP | PD |
| 6 | 62/F | Adenocarcinoma UO | N | HU/CARBOP | CR |
| 7 | 71/F | Adenocarcinoma UO | N | HU/CARBOP | PD |
| 8 | 73/M | Malignant melanoma | N | HU/DTIC | PR |
| 9 | 29/M | Malignant melanoma | N | HU/DTIC | PD |
| 10 | 65/F | Malignant melanoma | N | HU/DTIC | PD |
| 11 | 59/F | Malignant melanoma | Y | HU/CDDP | PD |
| 12 | 59/M | Malignant melanoma | N | HU/DTIC | PD |
| 13 | 64/M | Penile squamous cell ca | N | HU/CDDP | PD |
| 14 | 67/F | Angiosarcoma | Y | HU/CARBOP | PD |

[a]UO, unknown orign. [b]Chemo, chemotherapy; CDDP, cisplatin; CARBOP, carboplatin; DTIC, dacarbazine. [c]PD, progressive disease; CR, complete remission; PR, partial remission; SD, stable disease.

*Determination of the IUdR and Ki67 labelling indices (LI) in tumour and bone marrow aspirates*

A minimum of 500 malignant cells or BM precursor cells were counted and the percentage of tumour and bone marrow cells which were stained with either BU20a or Ki67 monoclonal antibodies calculated. Samples with too few cells were discarded.

*Determination of the percentage uptake of IUdR by cultured cells* in vitro *at increasing IUdR concentrations*

Cells from the human melanoma cell line A2058 which were in exponential growth were incubated with IUdR for 1 h. The final concentration of IUdR in the incubate ranged from 100 μM to 10 nM. Percentage of cells labelled with IUdR was determined as described above.

*Hydroxyurea plasma and ascitic fluid concentrations*

Ten ml of venous blood was collected from ten patients into a heparinised tube 0, 6 and 23 h from the start of the hydroxyurea infusion. Ten ml of ascitic fluid was also aspirated 23 h from the start of the hydroxyurea infusion in three patients. Plasma was immediately separated by centrifugation and stored at − 20°C pending analysis. The assay of hydroxyurea in plasma and ascites was undertaken using the standard colourimetric method described by Fabricus and Rajewsky (1971).

*Statistical analysis*

The paired two-tailed *t*-test was employed to compare two samples using StatView 512 + software package on an Apple Macintosh SE personal computer. Statistical significance was taken at a *P* value of ⩽ 0.05.

### Results

*Relationship between increasing concentration of IUdR and percentage of labelled A2058 cells*

Figure 1 shows the percentage of labelled cells over a range of IUdR concentrations between 100 μM and 10 nM. The maximum percentage of labelled cells was at concentrations of 10 μM and 100 μM. A fall in the IUdR concentration by at least two logs below 10 μM resulted in complete absence of staining with anti-IUdR antibodies.

**Table II** Hydroxyurea (HU) concentrations in plasma determined in ten patients, 6 and 23 h from the start of a continuous i.v. infusion at a rate of 1 g per h

| Patient | HU concentrations (mM) 6 h | 23 h |
|---|---|---|
| 2 | 1.33 | 1.87 (1.89) |
| 3 | 1.25 | 1.84 (1.96) |
| 6 | 0.89 | 1.37 |
| 7 | 0.87 | 2.13 (1.51) |
| 8 | 1.26 | 2.81 |
| 9 | 1.24 | 2.12 |
| 10 | 1.04 | 1.26 |
| 11 | 1.0 | 2.01 |
| 13 | 0.67 | 1.18 |
| 14 | 0.97 | 0.84 |
| Mean (± s.e.) | 1.1 ± 0.1 | 1.7 ± 0.2 |
| | *P* value <0.005 | |

Values in parentheses indicate concentration in ascitic fluid.

*Concentrations of hydroxyurea in plasma and ascitic fluid*

Mean (± s.e.) 6 and 23 h plasma hydroxyurea concentrations were 1.1 ± 0.1 and 1.7 ± 0.2 mM respectively (Table II). Concentrations at 23 h were higher ($P < 0.005$) than those achieved after 6 h of i.v. infusion and very similar to the 24 h concentration of hydroxyurea reported by Veale *et al.* (1988) using a similar rate of hydroxyurea infusion. Concentrations of hydroxyurea in ascitic fluid were comparable to those levels achieved in plasma (1.9, 2.0 and 1.5 mM) indicating adequate penetration of hydroxyurea into ascitic fluid.

In vitro *uptake of IUdR by tumour cells*

The mean (± s.e.) percentage of labelled cells by *in vitro* incubation with IUdR prior to any chemotherapy was similar in the non-hydroxyurea and hydroxyurea arms of the study (6.1 ± 1.6% and 5.9 ± 0.8% respectively, $P > 0.4$).

**Figure 1** Cells from A2058 cell line grown in the exponential phase were incubated with increasing concentrations of IUdR (0.01–100 μM). Values on the Y axis represent percentages of the maximum labelling index achieved in this experiment.

*Comparison of* in vitro *and* in vivo *uptake of IUdR by tumour cells*

In the group of patients not receiving hydroxyurea, incorporation of IUdR into tumour cells was determined *in vitro* before and *in vivo* after administering IUdR in order to assess whether there was any significant difference in the IUdR labelling index using either method. The percentage of *in vitro* IUdR labelled tumour cells (6.1 ± 1.6%) was not statistically different ($P > 0.1$) from the percentage of labelled cells following i.v. administration of IUdR (6.8 ± 1.1%) (Table III). The Ki67 LI of tumour cells before and after the administration of IUdR was again not significantly different (13.4 ± 2.9% and 14.1 ± 2.6%; $P > 0.4$).

In vivo *uptake of IUdR by tumour cells before and after hydroxyurea*

The mean ( ± s.e.) percentage of tumour cells labelled with BU20a in patients not treated with hydroxyurea was 6.8 ± 1.1% with a range of 4–13% (Table III). The mean ( ± s.e.) percentage of labelled tumour cells in the hydroxyurea treated group was 0.5 ± 0.4% (Table IV; $P < 0.0005$). There was detectable incorporation of IUdR in tumour cells from only three patients treated with hydroxyurea with a mean percentage of labelled cells of 2.4% and no detectable incorporation of IUdR (0%) in the rest of the patients. The IUdR labelling index did not change in one patient (No. 3) following treatment with hydroxyurea.

*Influence of hydroxyurea on Ki67 labelling index (LI) of tumour cells*

The mean ( ± s.e.) percentage of tumour cells labelled with Ki67 antibody determined prior to hydroxyurea administration and approximately 24 h from the start of the hydroxyurea infusion was similar (Table IV) (14.1 ± 1.5% and 13.0 ± 1.4% respectively, $P > 0.1$).

In vivo *incorporation of IUdR and Ki67 labelling index of bone marrow cells before and after hydroxyurea*

The mean ( ± s.e.) percentage of marrow prescursor cells taking up IUdR in ten patients who did not receive hydroxyurea was 12.2 ± 1.8% with a range of 2–22% (Table III), whereas in the nine patients who were treated with hydroxyurea the mean percentage of labelled bone marrow precursor cells was significantly reduced (0.1 ± 0.1%, $P < 0.0005$) (Table IV). BM aspiration was not feasible from 3 patients and the samples from another three patients were not satisfactory. The Ki67 labelling index was slightly reduced following treatment with hydroxyurea (18.8% *vs* 15.6% respectively, $P > 0.05$).

**Table III** IUdr and Ki67 labelling index of tumour and bone marrow aspirates determined in 11 patients following treatment with i.v. IUdR 100 mg m$^{-2}$ only

| | *Labelling index (%)* | | | | | |
|---|---|---|---|---|---|---|
| | *Pre-IUdR/Tumour (in vitro)* | | *Post-IUdR/Tumour (in vivo)* | | *Post-IUdR/BM (in vivo)* | |
| No | Ki67 | BU20a | Ki67 | BU20a | Ki67 | BU20 |
| 15 | ND | ND | ND | ND | 18.0 | 14.5 |
| 16 | ND | ND | ND | ND | 19.5 | 9.0 |
| 17 | 12.5 | 7.0 | 11.0 | 6.5 | 25.0 | 22.0 |
| 18 | 18.0 | 9.9 | 16.5 | 13.0 | 10.0 | 2.0 |
| 19 | 5.0 | 2.0 | 15.0 | 7.0 | 24.0 | 10.0 |
| 20 | 14.5 | 5.5 | 10.0 | 5.5 | 17.0 | 16.0 |
| 21 | NC | NC | NC | NC | 19.0 | 16.0 |
| 22 | 27.5 | 13.0 | 25.0 | 7.0 | 17.0 | 6.0 |
| 23 | 6.0 | 2.5 | 7.0 | 4.0 | 20.0 | 15.0 |
| 24 | 10.0 | 3.0 | NC | 4.5 | ND | ND |
| 25 | NC | NC | NC | NC | 18.0 | 11.0 |
| Mean ( ± s.e.) | 13.4 ± 2.9 | 6.1 ± 1.6 | 14.1 ± 2.6 | 6.8 ± 1.1 | 18.8 ± 1.3 | 12.2 ± 1.8 |

ND, aspiration not performed; NC, inadequate cell count. Pre-treatment tumour samples were also obtained and incubated with IUdR 10 μM.

**Table IV** IUdR and Ki67 labelling index of tumour and bone marrow (BM) aspirates in 14 patients who received a continuous i.v. infusion of hydroxyurea (1 g per h)

| | *Labelling index (%)* | | | | | |
|---|---|---|---|---|---|---|
| | *Pre-HU/Tumour (in vitro)* | | *Post-HU/Tumour (in vivo)* | | *Post-HU/BM (in vivo)* | |
| No | Ki67 | BU20a | Ki67 | BU20a | Ki67 | BU20 |
| 1 | 12.2 | 6.5 | 10.0 | 0 | 10.0 | 0 |
| 2 | 11.0 | 1.5 | 8.0 | 0 | NC | NC |
| 3 | 9.5 | 4.9 | 10.0 | 5 | ND | ND |
| 4 | 21.0 | 10.0 | 20.0 | 0 | 22.0 | 0 |
| 5 | 15.0 | 10.0 | 10.0 | 2.0 | 25.0 | 0 |
| 6 | 18.0 | 9.9 | 15.0 | 0 | 18.0 | 0 |
| 7 | ND | ND | 20.0 | 0 | NC | NC |
| 8 | 12.5 | 6.0 | PS | 0 | 10.0 | 0 |
| 9 | 14.0 | 4.0 | 9.0 | 0 | 14.0 | 0 |
| 10 | 17.0 | 5.0 | 18.0 | 0.1 | 16.0 | 0 |
| 11 | 24.2 | 4.7 | 15.0 | 0 | 15.0 | 0 |
| 12 | 7.0 | 3.0 | 5.0 | 0.0 | ND | ND |
| 13 | ND | ND | 18.5 | 0 | NC | NC |
| 14 | 9.5 | 5.0 | 10.0 | 0 | 10.0 | 0.5 |
| Mean ( ± s.e.) | 14.2 ± 1.5 | 5.9 ± 0.8 | 13.0 ± 1.4 | 0.5 ± 0.4 | 15.6 ± 1.8 | 0.1 ± 0.1 |

ND, aspiration not performed; NC, inadequate cell count; PS, poor staining. Pre-treatment tumour aspirates were also obtained and incubated with IUdR 10 μM for 1 h.

*Response to treatment*

Details of tumour response to hydroxyurea based chemotherapy are shown in Table I. Patients received a mean number of 3.3 courses with a range of 1–6. Standard criteria were applied to determine response to treatment. One patient with ovarian adenocarcinoma with ascites and an abdominal mass and another with adenocarcinoma of unknown origin presenting with ascites achieved durable (10 months and 19 months) complete remission after receiving six courses of hydroxyurea with cisplatin and hydroxyurea with carboplatin respectively. One other patient with malignant melanoma achieved a partial remission in cutaneous metastases which lasted for approximately 4 months.

### Discussion

This study shows that hydroxyurea significantly inhibited the IUdR incorporation by bone marrow and tumour cells through the inhibition of DNA synthesis. Mean plasma and ascitic fluid hydroxyurea concentrations of greater than 1 mM were achieved which were similar to effective inhibitory concentrations in appropriate *in vitro* models. Belt *et al.* (1980) described an intravenous schedule delivering a lower rate (approximately 1/3) of hydroxyurea compared to the present study and showed that there was no appreciable effect on the uptake of radiolabelled thymidine in bone marrow and tumour cells after 72 h of continuous infusion. It is probable that their technique of *in vitro* incubation with radiolabelled thymidine for the estimation of the S-phase washed out the hydroxyurea from cells resulting in the removal of its inhibitory effect. In our study, hydroxyurea significantly inhibited the synthesis of DNA in tumour cells despite the fact that mean cell-cycle times in those cells are expected to be significantly longer (Wilson *et al.*, 1988) than the 23 h exposure period to hydroxyurea. Threshold cytostatic and cytotoxic concentrations have been suggested (Timson, 1969) and it has been shown that a concentration of 1 mM of hydroxyurea will effectively inhibit DNA synthesis in most experimental systems (Sinclair, 1965).

The optimum concentration of IUdR for *in vitro* incubation (10 $\mu$M) was based on the predicted range of plasma concentrations of IUdR (Hoshino *et al.*, 1985). The percentage of cells labelled by IUdR in the A2058 cell line was shown to be constant at concentrations of 10 $\mu$M and 100 $\mu$M. A 2-log decrease in IUdR concentration resulted in complete loss of immunocytochemical detection of IUdR incorporation by those cells. It may thus be infered that the degree of DNA synthesis inhibition detected in the majority of tumour cells and bone marrow following treatment with hydroxyurea was at least in the order of 2 logs.

Previous clinical trials have concluded that solid tumours respond poorly to hydroxyurea. Consequently hydroxyurea, especially as single agent, is not included in the conventional systemic therapy of such tumours. The principal biochemical target of hydroxyurea is ribonucleotide reductase which is a key enzyme in *de novo* DNA synthesis. Altered expression of ribonucleotide reductase due to gene amplification has been suggested as a mechanism of resistance to hydroxyurea in certain cell lines (Wright, 1987). This study was designed to assess *in vivo* biochemical effects of high dose hydroxyurea and most patients had tumours which were considered resistant to conventional chemotherapy. IUdR incorporation was effectively suppressed in 11 of the 14 cases treated with hydroxyurea but only three of the 14 patients showed objective evidence of tumour response.

Bagshawe *et al.* (1987) demonstrated that hydroxyurea pre-treatment of mice bearing human tumour xenografts which were known to be resistant to hydroxyurea resulted in a reduction of the uptake of radiolabelled IUdR by normal cells without a concomitant decrease in the incorporation of IUdR by the tumours cells. The results of our study show that hydroxyurea inhibits the incorporation of IUdR by the tumour cells indicating that clinical resistance to hydroxyurea should not be equated with resistance to hydroxyurea at the cellular level. Determination of biochemical parameters, such as the IUdR labelling index, will therefore not predict the eventual outcome of treatment with hydroxyurea or other similar drugs.

The schedule of hydroxyurea administration in our study was different from that employed by Bagshawe *et al.* which employed bolus administrations of hydroxyurea rather than continuous infusion. In their study uptake of IUdR was determined by autoradiography using [$^{125}$I]-labelled IUdR. The different outcome between the two studies is probably due to the variable drug susceptibility between different tissues as a consequence of different threshold values of hydroxyurea plasma inhibitory concentrations to tumour and BM cells. It is also possible that tumour xenografts investigated by Bagshawe *et al.* (1987) were more resistant to hydroxyurea than the tumours in our study.

The higher values for the Ki67 labelling index compared to the percentage labelled cells with BU20a is because Ki67 antigen is expressed in cells throughout the cell cycle whereas IUdR labelled cells are those only in the S-phase. The absence of any significant change in Ki67 labelling index of tumour and BM cells is compatible with the synchronisation of the cells by hydroxyurea at the $G_1$/S interphase. This is explained by the fact that Ki67 antigen is expressed by all phases of the cell cycle and hydroxyurea treated cells will be blocked at $G_1$/S interphase. Ki67 labelling will therefore be not helpful in studying cell kinetics following treatment with antimetabolites.

This work however shows that it is possible to study *in vivo* the acute influence of systemic treatment on DNA synthesis in normal and tumour cells. This may provide an excellent model for insight into the pharmacological action of those drugs allowing the design of rational schedules of therapy. Conventional methods of bone marrow aspiration are not routinely incorporated into research protocols because of the degree of discomfort incurred on patients. The procedure used in this study resulted in little or no discomfort to patients because of the small needle size. The use of immunocytochemistry to obtain information regarding the S-phase fraction and growth fraction of tumour and bone marrow may provide a quick method to monitor therapy and modify treatment within a reasonably short space of time.

Hydroxyurea effectively inhibits DNA synthesis in tumour cells *in vivo* and based on the results of uptake of IUdR in the A2058 cell line it can be assumed that $>99\%$ inhibition in DNA synthesis was seen in bone marrow and tumour cells. It is possible that employing a lower level of inhibition of hydroxyurea may favourably produce a differential inhibition of DNA synthesis in BM and tumour cells and allow the selective uptake of IUdR by tumour cells. Nevertheless, it was possible to achieve plasma concentrations of hydroxyurea close to levels which are inhibitory to DNA repair *in vitro* (Snyder, 1984). This study forms the basis of our current schedule of hydroxyurea to inhibit repair of cisplatin-DNA adducts using a loading schedule of administration followed by a longer maintenance infusion relative to the administration of the DNA damaging agent.

### References

ARIEL, I.M. (1969). Therapeutic effects of hydroxyurea. Experience with 118 patients with inoperable solid tumours. *Cancer*, **25**, 705–714.

BAGSHAWE, K.D. (1986). Reversed-role chemotherapy for resistant cancer. *Lancet*, **ii**, 778–780.

BAGSHAWE, K.D., BODEN, J., BOXER, G.M., BRITTON, D.W., GREEN, A., PARTRIDGE, T., PEDLEY, B., SHARMA, S. & SOUTHALL, P. (1987). A cytotoxic DNA precursor is taken up selectively by human cancer xenografts. *Br. J. Cancer*, **55**, 299–302.

BEGG, A.C., MOONEN, L., HOFLAND, I., DESSING, M. & BARTELINK, H. (1988). Human tumour cell kinetics using a monoclonal antibody against iododeoxyuridine: intratumour sampling variations. *Radiotherapy Oncology*, **11**, 337–347.

BELT, R.J., HAAS, C.D., KENNEDY, J. & TAYLOR, S. (1980). Studies of hydroxyurea administered by continuous infusion. *Cancer*, **46**, 455–462.

BLOOMER, W.D. & ADELSTEIN, S.J. (1978). 5-($^{125}$I)-iododeoxyuridine and the auger effect: biological consequences and implications for therapy. In Ioachim, H.L. (ed.), *Pathobiology Annual*, Vol. 8, pp. 407–421. New York: Raven Press.

CALABRESI, P., CARDOSO, S.S., FINCH, S.C., KLIGERMAN, M.M., VON ESSEN, C.F., CHU, M.Y. & WELCH, A.D. (1961). Initial clinical studies with 5-iodo-2-deoxyuridine. *Cancer Res.*, **21**, 550–559.

CORDELL, J.L., FALINI, B., ERBER, W.N., GHOSH, A.K., ABDULAZIZ, Z., MACDONALD, S., PULFORD, K.A., STEIN, H. & MASON, D.Y. (1984). Immunoenzymatic labelling of monoclonal antibodies using immune complexes of alkaline phosphatase and monoclonal anti-alkaline phosphatase (APAAP) complexes. *J. Histochem. Cytochem.*, **32**, 219–229.

FABRICUS, E. & RAJEWSKY, M.F. (1971). Determination of hydroxyurea in mammalian tissues and blood. *Rev. Europ. Etud. Clin. Et. Biol.*, **16**, 679–683.

FORNACE, A.J., DOBSON, P.P. & KINSELLA, T.J. (1990). Enhancement of radiation damage in cellular DNA following unifilar substitution with iodo-deoxyuridine. *Int. J. Radiation Oncol. Biol. Phys.*, **18**, 873–878.

GERDES, J. (1985). An immunohistological method for estimating cell growth fractions in rapid histopathological diagnosis during surgery. *Int. J. Cancer*, **35**, 169–171.

GOLDIE, J.H. & COLDMAN, A.J. (1984). Genetic instability in the development of drug resistance. *Sem. Oncol.*, **12**, 222–230.

HOSHINO, T., NAGASHIMA, T., MUROVIC, J., LEVIN, E.M., LEVIN, V.A. & RUPP, S. (1985). Cell kinetic studies of an in situ human brain tumours with bromodeoxyuridine. *Cytometry*, **6**, 627–632.

KAMATA, T., YONEMURA, Y., SUGIYAMA, K., OOYAMA, S., KOSAKA, T., YAMAGUCHI, A., MIWA, K. & MIYAZAKI, I. (1989). Proliferative activity of early gastric cancer measured by *in vitro* and *in vivo* bromodeoxyuridine labeling. *Cancer*, **64**, 1665–1668.

KAUNG, D.T., WALSH, W.S., SBAR, S. & PATNO, M.E. (1968). Hydroxyurea (NSC-32065) in therapy for nonresectable cancer of the lung. *Cancer Chemother. Reports*, **52**, 271–274.

KINSELLA, T.J., RUSSO, A., MITCHELL, J.B., COLLINS, J.M., ROWLAND, J., WRIGHT, D. & GLATSTEIN, E. (1985). A phase I study of intravenous iododeoxyuridine as a clinical radiosensitizer. *Int. J. Radiation Oncol. Biol. Phys.*, **11**, 1941–1946.

LEWIS, W.H. & WRIGHT, J.A. (1974). Altered ribonucleotide reductase activity in mammalian tissue culture cells resistant to HU. *Biochem. Biophysic. Res. Commun.*, **60**, 926–933.

PLAGMANN, P.G.W. & ERBE, J. (1974). Intracellular conversions of deoxyribonucleosides by Novikoff rat hepatoma cells and effects of hydroxyurea. *J. Cell Physiol.*, **83**, 321–336.

SINCLAIR, W.K. (1965). Hydroxyurea: differential lethal effects on cultured mammalian cells during the cell cycle. *Science*, **150**, 1729–1731.

SNYDER, R.D. (1984). Inhibitors of ribonucleotide reductase alter DNA repair in human fibroblasts through specific depletion of purine deoxynucleotide triphosphates. *Cell Biol. Toxicol.*, **1**, 81–94.

SNYDER, R.D., VAN HOUTEN, B. & REGAN, J.D. (1984). The accumulation of DNA breaks due to incision; comparative studies with various inhibitors. In Collins, A., Downes, C.S. & Johnson, R.T. (eds). *DNA Repair and its Inhibition*. pp. 13–33. Oxford, England: IRL Press.

SPETH, P.A., KINSELLA, T.J., CHANG, A.E., KLECKER, R.W., BELANGER, K., SMITH, R., ROWLAND, J., CUPP, J.E. & COLLINS, J.M. (1989). Iododeoxyuridine (IdUrd) incorporation into DNA of human hematopoietic cells, normal liver and hepatic metastases in man: as a radiosensitizer and as a marker for cell kinetic studies. *Int. J. Radiation Oncology Biol. Phys.*, **16**, 1247–1250.

TIMSON, J. (1969). Hydroxyurea: comparison of cytotoxic and antimitotic activities against human lymphocytes *in vitro*. *Br. J. Cancer*, **23**, 337–339.

VEALE, D., CANTWELL, B.M.J., KERR, N., UPFOLD, A. & HARRIS, A.L. (1988). Phase I study of high-dose hydroxyurea in lung cancer. *Cancer Chemother. Pharmacol.*, **21**, 53–56.

WILSON, G.D., MCNALLY, N.J., DISCHE, S., SAUNDERS, M.I., DESROCHERS, C., LEWIS, A.A. & BENNETT, M.H. (1988). Measurement of cell kinetics in human tumours *in vivo* using bromodeoxyuridine incorporation and flow cytometry. *Br. J. Cancer*, **58**, 423–431.

WRIGHT, J.A., ALAM, T.G., MCCLARTY, G.A., TAGGER, A.Y. & THELANDER, L. (1987). Altered expression of ribonucleotide reductase and role of M2 gene amplification in hydroxyurea-resistant hamster, mouse, rat and human cell lines. *Somatic Cell Molecular Genetics*, **13**, 155–165.